# Association between Ambient Particulate Air Pollution and Soluble Biomarkers of Endothelial Function: A Meta-Analysis

**DOI:** 10.3390/toxics12010076

**Published:** 2024-01-15

**Authors:** Kai Wang, Lei Lei, Ge Li, Yang Lan, Wanzhou Wang, Jiaqi Zhu, Qisijing Liu, Lihua Ren, Shaowei Wu

**Affiliations:** 1 Department of Occupational and Environmental Health, School of Public Health, Xi’an Jiaotong University Health Science Center, Xi’an 710061, China; wk10716@163.com (K.W.); leilei981118@163.com (L.L.); yang.lan@xjtu.edu.cn (Y.L.); jqzhuu@gmail.com (J.Z.); 2 Key Laboratory for Disease Prevention and Control and Health Promotion of Shaanxi Province, Xi’an 710061, China; 3 Key Laboratory of Trace Elements and Endemic Diseases in Ministry of Health, Xi’an 710061, China; 4 Key Laboratory of Environment and Genes Related to Diseases, Xi’an Jiaotong University, Ministry of Education, Xi’an 710061, China; 5 Department of Occupational and Environmental Health Sciences, School of Public Health, Peking University, Beijing 100191, China; 1610306235@pku.edu.cn; 6 Research Institute of Public Health, School of Medicine, Nankai University, Tianjin 300071, China; liuqisijing@126.com; 7 School of Nursing, Peking University, Beijing 100191, China; renlihua@bjmu.edu.cn

**Keywords:** ambient air pollution, endothelial function, intercellular adhesion molecule-1, vascular cell adhesion molecule-1, endothelin-1, E-selectin

## Abstract

Background: The burden of cardiovascular diseases caused by ambient particulate air pollution is universal. An increasing number of studies have investigated the potential effects of exposure to particulate air pollution on endothelial function, which is one of the important mechanisms for the onset and development of cardiovascular disease. However, no previous study has conducted a summary analysis of the potential effects of particulate air pollution on endothelial function. Objectives: To summarize the evidence for the potential effects of short-term exposure to ambient particulate air pollution on endothelial function based on existing studies. Methods: A systematic literature search on the relationship between ambient particulate air pollution and biomarkers of endothelial function including endothelin-1 (ET-1), E-selectin, intercellular cell adhesion molecule-1 (ICAM-1), and vascular cell adhesion molecule-1 (VCAM-1) was conducted in PubMed, Scopus, EMBASE, and Web of Science up to 20 May 2023. Subsequently, a meta-analysis was conducted using a random effects model. Results: A total of 18 studies were included in this meta-analysis. A 10 μg/m^3^ increase in short-term exposure to ambient PM_2.5_ was associated with a 1.55% (95% CI: 0.89%, 2.22%) increase in ICAM-1 and a 1.97% (95% CI: 0.86%, 3.08%) increase in VCAM-1. The associations of ET-1 (0.22%, 95% CI: −4.94%, 5.65%) and E-selectin (3.21%, 95% CI: −0.90% 7.49%) with short-term exposure to ambient PM_2.5_ were statistically insignificant. Conclusion: Short-term exposure to ambient PM_2.5_ pollution may significantly increase the levels of typical markers of endothelial function, including ICAM-1 and VCAM-1, suggesting potential endothelial dysfunction following ambient air pollution exposure.

## 1. Introduction

With the continuous improvement of global industrialization, emissions from automotive exhausts, industrial emissions, garbage incineration, cigarette smoke and other sources of air pollutants have emerged [1]. Air pollution has become one of the most important environmental factors affecting public health. Previous studies have shown that particulate air pollution may cause more substantial health damage than gaseous air pollutants [2], particularly fine particulate matter (PM_2.5_, particulate matter with an aerodynamic diameter ≤ 2.5 μm). According to the Global Burden of Disease Assessment [3], exposure to air pollution is responsible for about 7 million premature deaths and more than 3% of disability-adjusted life years lost worldwide [4]. Even low concentrations of PM_2.5_ (below the European limit value of 25 μg/m^3^) have been associated with a 13% increase in non-fatal acute coronary events [5]. There is consistent evidence that both short- and long-term exposures to particulate air pollution are associated with cardiovascular disease morbidity and mortality [2,6,7]. Cardiovascular disease is responsible for more than two-thirds of fatal illnesses linked to particulate air pollution [8].

Particulate matter is classified by size into inhalable particulate matter (PM_10_, particulate matter with an aerodynamic diameter ≤ 10 μm), PM_2.5_, and ultrafine particles (UFPs, with an aerodynamic diameter ≤ 0.1 μm) [9,10]. Particulate matter is composed of complex organic molecules, metal elements and other inorganic ions such as sulfate radicals and nitrate radicals. These particles originate from various sources such as automobile emissions, industrial processes, and daily living environment emissions [11,12].

Particulate matter exposure can induce the development of cardiovascular diseases through various mechanisms, including systemic inflammation, endothelial dysfunction, direct toxicity of the cardiovascular system, mitochondrial dysfunction, autophagy, apoptosis and oxidative stress damage [13,14,15,16,17]. When endothelial dysfunction occurs, it disrupts the body’s anticoagulant process and interferes with vascular repair [18]. Deterioration of vascular function due to endothelial dysfunction is a key predictor of cardiovascular disease due to its important role in the development of atherosclerosis [19]. Previous studies have shown that markers of endothelial function in people with cardiovascular disease are associated with exposure to air pollution [20,21]. Although there has been some epidemiological evidence in this area, a meta-analysis is still lacking to synthesize the evidence and provide a basis for future research. 

Various endothelial function factors have been used as research objects, such as endothelin-1 (ET-1), E-selectin, intercellular adhesion molecule-1 (ICAM-1) and vascular cell adhesion molecule-1 (VCAM-1) [22]. ET-1, E-selectin, ICAM-1, and VCAM-1, expressed in various disease states associated with endothelial dysfunction, serve as reliable markers of inflammation and endothelial dysfunction, playing crucial roles in the pathophysiology of cardiovascular diseases. ICAM-1 and VCAM-1 are expressed on the cell surface and serve as cell adhesion molecules to mediate the transformation of monocytes and eosinophils into vascular endothelial cells, which exist in a soluble form in the plasma and are reliable markers of inflammation and endothelial dysfunction [23,24]. Studies have shown that the adhesion of monocytes to activated endothelial cells and subsequent migration to the vascular wall are key to the development of atherosclerosis [25].

There is currently no specific meta-analysis on the association between particulate air pollution and individual endothelial function markers. Therefore, this meta-analysis was conducted to consolidate existing research findings by selecting four representative endothelial function markers for a summary analysis of the impact of short-term and long-term exposures to particulate air pollution on endothelial function. 

## 2. Materials and Methods

### 2.1. Search Strategy

To search for articles, we performed a comprehensive search using relevant keywords of exposure and outcomes in the following databases: PubMed (all fields), EMBASE (all fields), Scopus (article title, abstract and keywords) and Web of Science (topic) up to 20 May 2023. The terms related to ambient PM and “ICAM-1”, “VCAM-1”, “ET-1”, and “E-selectin” and their synonyms were used in searches. The detailed retrieval strategies for this meta-analysis are provided in the Supplementary Materials, Table S1.

### 2.2. Study Selection Criteria

We examined all papers by title, abstract, or full text by using inclusion and exclusion criteria. Inclusion criteria: epidemiological studies that provided quantitative estimates of the associations between ambient particulate matter and endothelial function biomarkers in adults; when multiple studies were conducted on the same set of subjects, we selected the most recent one.

Exclusion criteria: studies that investigated indoor exposures or occupational exposures instead of outdoor/ambient air pollution; studies that did not or failed to quantify the associations of interest; non-epidemiological studies such as in vitro or in vivo studies, phytological studies, or toxicological studies; and review articles, editorials, commentaries, conference proceedings, and case reports. Articles without available data were excluded if there was no response from the authors to our requests for information.

### 2.3. Data Extraction

Two reviewers (L. Lei and K. Wang) independently screened the retrieved records and extracted data. Two investigators conducted further reviews by screening the abstracts and titles identified in the preliminary survey. Then, all full-text articles that might have met the criteria for data extraction were reviewed. If a disagreement appeared over the review, the disagreement was resolved via preliminary discussion between the two investigators, and a decision was made by a third investigator (S. Wu). We extracted the study data and characteristics of all included studies, including the following: (1) citation information (title, author, journal and year of publication), (2) study period, (3) study location, (4) participant characteristics (amount, age, the ratio of female, disease status), (5) study design, (6) the concentration of exposure (PM_2.5,_ PM_10_), (7) the concentration of biomarkers (ET-1, E-selectin, ICAM-1, VCAM-1), (8) measurement methodology of exposure, and (9) effect estimates, a unit of the effect estimates and 95% confidence intervals (CIs). For articles lacking data that could be directly extracted, we contacted the corresponding author of the article to obtain data. The estimated effects were categorized into short-term effects lasting a few weeks or days and long-term effects lasting more than six months [26]. During data extraction, for all qualified articles, we extracted and recorded the exposure metrics. If multiple lag times were reported in the article, we selected the largest effect estimate with statistical significance. When none of the effect estimates were significant, we selected the effect estimate with the smallest *p* value if *p* values were reported and the largest effect estimate if no *p* values were reported. If multiple effect models were included, we extracted the single pollution effect model or the model adjusted for the largest number of covariates.

All included studies were assessed for quality using the Effective Public Health Practices Project (EPHPP) tool. This quality evaluation tool fully considers the characteristics of the articles, such as experimental design, population sources, etc. The details of EPHPP can be seen in the Supplementary Materials, Table S2. The quality evaluation work was carried out independently by two investigators (L. Lei and K. Wang). If there was any difference, the third investigator (S. Wu) would resolve it.

### 2.4. Statistical Analysis

We used percent change (%) as the association measure for all included studies. For consistency, all effect estimates were converted to percent changes in endothelial function biomarkers per 10 g/m^3^ exposure to ambient PM_2.5_ and PM_10_. In this meta-analysis, the results reported in different forms in the included studies were considered to be equivalent to the percent changes in effect estimates multiplied by 100%. We performed an antilog transformation on log-transformed data. By subtracting one from the fold change of biomarkers, and then multiplying by 100%, we obtained the pattern of percent change in biomarkers. We used [β ×10 ÷ M] × 100% to convert regression coefficients into percent changes in endothelial function biomarkers per 10 μg/m^3^ exposure to ambient PM_2.5_ in results that were not log-transformed, where β represents the regression coefficient and M represents the mean level of the target biomarker. Furthermore, 95% CIs were calculated using the following formula [(β ± 1.96 × SE) × 10 ÷ M] × 100%, and SE represents the standard error of β [27].

Percent changes and 95% CIs of the biomarkers in all the included studies were summarized using a random effects model. To assess the heterogeneity among the included studies, we employed the I-squared statistic and the chi-square-based Cochran Q statistic test, and I^2^ > 50% and *p* value < 0.10 were considered to indicate statistically significant heterogeneity [28].

The number of studies reporting associations between long-term (*n* = 1) and short-term (*n* = 2) exposures to ambient PM_10_ and short-term exposures to PM_2.5_ (*n* = 1) and endothelial function biomarkers was limited. Therefore, we only performed meta-analyses for the associations of short-term exposure to ambient PM_2.5_ and endothelial function biomarkers.

We then performed subgroup analyses for the results with significant heterogeneity, including area, sample size, age, participant, female proportion, exposure assessment, study design, and quality of study. We also performed sensitivity analyses for the pooled effect estimates by removing one study each at a time to assess the effect of the excluded study on the pooled results. A funnel plot, as an adjunct to a forest plot, is commonly used to evaluate publication bias for the inclusion of studies with numbers close to 10 [29]. Egger’s and Begg’s tests were also constructed to explore potential publication bias. The trim-and-fill method is a nonparametric approach used to adjust the asymmetry of the funnel plot and to investigate the number of missing studies [30,31]. We used these approaches to analyze the publication bias for the associations between PM_2.5_ and ICAM-1 and VCAM-1. All of the above results were calculated using R statistical software (R version 4.1.3), and the statistical significance was defined as *p* < 0.05 (2-sided), except that *p* for heterogeneity <0.10 was used for the meta-analyses of all studies and subgroup difference tests.

## 3. Results

**Figure 1:**
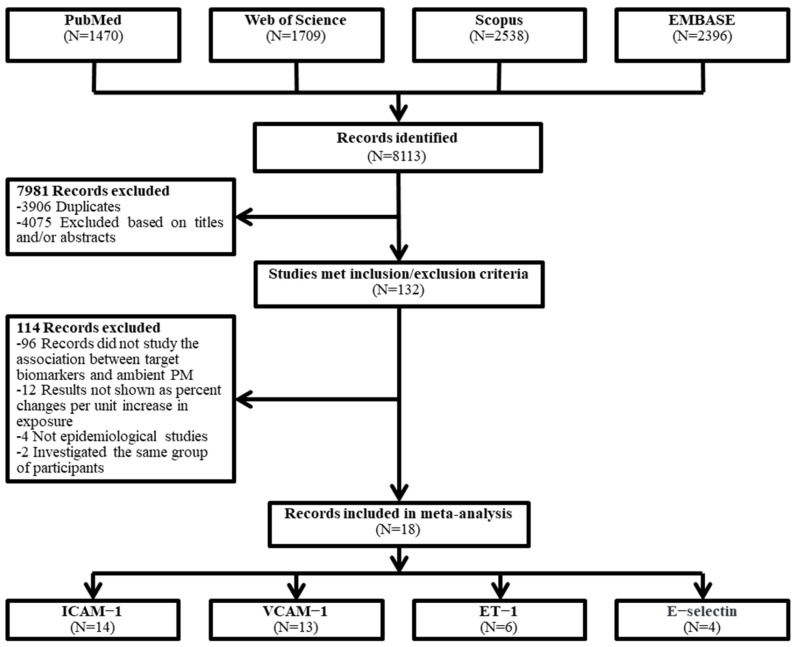
Flowchart of the literature search for the meta-analysis.

As shown in Figure 1, 8113 potentially relevant articles were selected from four databases through keyword retrieval, and then 18 studies with a total of 9611 participants were eventually included in this meta-analysis through strict exclusion and inclusion criteria [15,21,32,33,34,35,36,37,38,39,40,41,42,43,44,45,46,47], including 14 studies for ICAM-1, 13 studies for VCAM-1, 6 studies for ET-1, and 4 studies for E-selectin. The quality of the included studies was evaluated via the EPHHP, and the quality of the studies remained generally good or moderate (Table S3). The details of the article are shown in Table 1.

Although we searched for both long-term and short-term exposures to ambient PM_10_ and PM_2.5_ in the literature retrieval, the number of eligible studies was too few to conduct a summary analysis for long-term exposure to PM_10_ (*n* = 1) and PM_2.5_ (*n* = 1) and short-term exposure to PM_10_ (*n* = 2).

**Figure 2:**
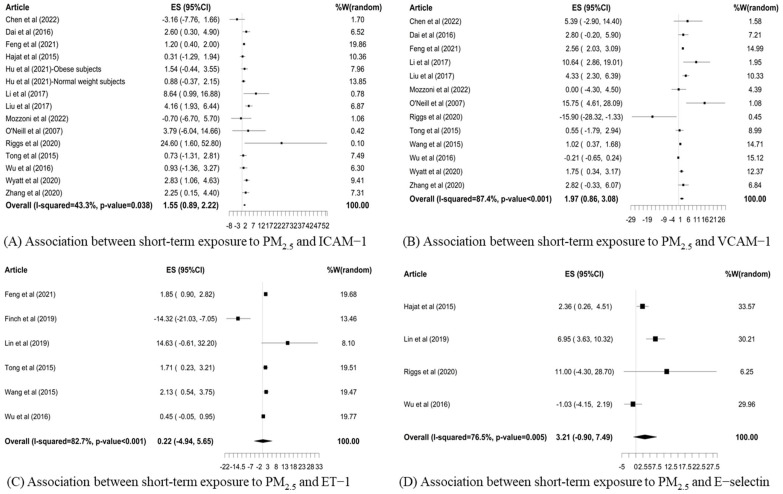
Forest plots [15,21,32,33,34,35,36,37,38,39,40,41,42,43,44,45,46,47] of pooled percent changes (%) and 95% confidence intervals (CIs) in the biomarkers of endothelial function in association with a 10 μg/m^3^ increase in short-term exposure to PM_2.5_.

The forest plots (Figure 2) showed that the pooled percent changes of endothelial function biomarkers per 10 μg/m^3^ increase in short-term exposure to ambient PM_2.5_ were 1.55% (95% CI: 0.89%, 2.22%) in ICAM-1 and 1.97% (95% CI: 0.86%, 3.08%) in VCAM-1. The estimated percent changes in endothelial function biomarkers per 10 μg/m^3^ increase in measured PM_2.5_ were 0.22% (95% CI: −4.94%, 5.65%) in ET-1 and 3.21% (95% CI: −0.90%, 7.49%) in E-selectin, respectively. Meanwhile, the degree of heterogeneity was mild for the pooled estimates for ICAM-1 and E-selectin and moderate to high for VCAM-1 and ET-1.

No statistically significant heterogeneity was observed among subgroups for ICAM-1 and VCAM-1 (Table 2 and Table 3). Because there were only a few studies about ET-1 and E-selectin that met the inclusion criteria, we did not perform subgroup analysis for ET-1 and E-selectin.

Sensitivity analyses (Table S4) revealed that the effect estimates were stable for ICAM-1, VCAM-1, suggesting that no single study influenced the overall pooled results. For ET-1, the pooled effect estimate was different after omitting the study conducted by [35], and the test result of heterogeneity also changed, possibly because there were fewer included studies for ET-1. Funnel plots were used to detect publication bias, which showed slight asymmetry for ICAM-1 and VCAM-1 (Figures S1 and S2). Both Egger’s (*p* = 0.213) and Begg’s tests (*p* = 0.125) for ICAM-1 indicated no publication bias (Table S5). Similarly, for VCAM-1, the results of Egger’s (*p* = 0.290) and Begg’s tests (*p* = 1.000) verified that the publication bias was insignificant. As shown in Figures S1 and S2, unpublished studies with negative results need to be added to the non-statistically significant area (white area) to balance the asymmetry of the funnel plot, indicating the existence of publication bias.

## 4. Discussion

### 4.1. Main Findings and Differences Compared with Other Similar Reviews 

We conducted a systematic review and meta-analysis on the existing epidemiologic evidence to assess whether short-term exposure to ambient PM_2.5_ was associated with major biomarkers of endothelial function, including ICAM-1, VCAM-1, ET-1 and E-selectin. For these four markers of endothelial function, our results showed that a 10 μg/m^3^ increase in short-term exposure to ambient PM_2.5_ was associated with significant increases of 1.55% and 1.97% in ICAM-1 and VCAM-1, respectively. However, no statistically significant results were found for ET-1 and E-selectin. The results of the subgroup analysis showed no statistically significant heterogeneity among studies. However, our meta-analysis obtained some insignificant associations between particulate air pollution and biomarkers of endothelial function, which may be due to the limited number of studies available for the meta-analysis.

To our knowledge, this is the first study to explore the association between short-/long-term exposure to ambient PM_2.5_ and major biomarkers of endothelial function in the blood. A recent meta-analysis on a similar topic has focused on vascular function indicators, including flow-mediated dilation, reactive hyperemia index, pulse wave velocity and augmentation index, but not biomarkers in the blood [48]. However, the circulating biomarkers reflecting endothelial dysfunction, as an important influencing factor of cardiovascular diseases, have not been comprehensively analyzed concerning particulate air pollution in previous studies. Biomarkers of endothelial dysfunction with good specificity and sensitivity have emerged as potential diagnostic or predictive factors for cardiovascular disease [49]. Meanwhile, as endothelial activation precedes and may stimulate the development of atherosclerotic lesions, the measurement for biomarkers of endothelial activation in the context of atherosclerosis is of great importance [50,51]. In a previous meta-analysis on particulate matter exposure and clotting markers, E-selectin and ICAM-1 were regarded as indirect clotting markers and combined with sera soluble CD40L, *p*-selectin and plasminogen activator inhibitor type-1 in the analysis; it was found that a per 5000/cm^3^ increase in UFPs exposure corresponded to a combined effect estimate of 10.83% (95% CI: 3.49%, 18.17%) [52]. However, it is not reliable to extend this conclusion to the analysis on the associations between particulate air pollution and individual biomarkers of endothelial function due to different inclusion purposes, different index measurement methods and the combined analysis of multiple indexes. Overall, our meta-analysis provides a comprehensive, in-depth, and precise assessment of the evidence focusing on ambient PM_2.5_ exposure as a potential cause of endothelial dysfunction. It could inform the focus of future research and is a timely contribution to a rapidly evolving field.

### 4.2. Underlying Mechanisms Related to Targeted Molecules 

**Figure 3:**
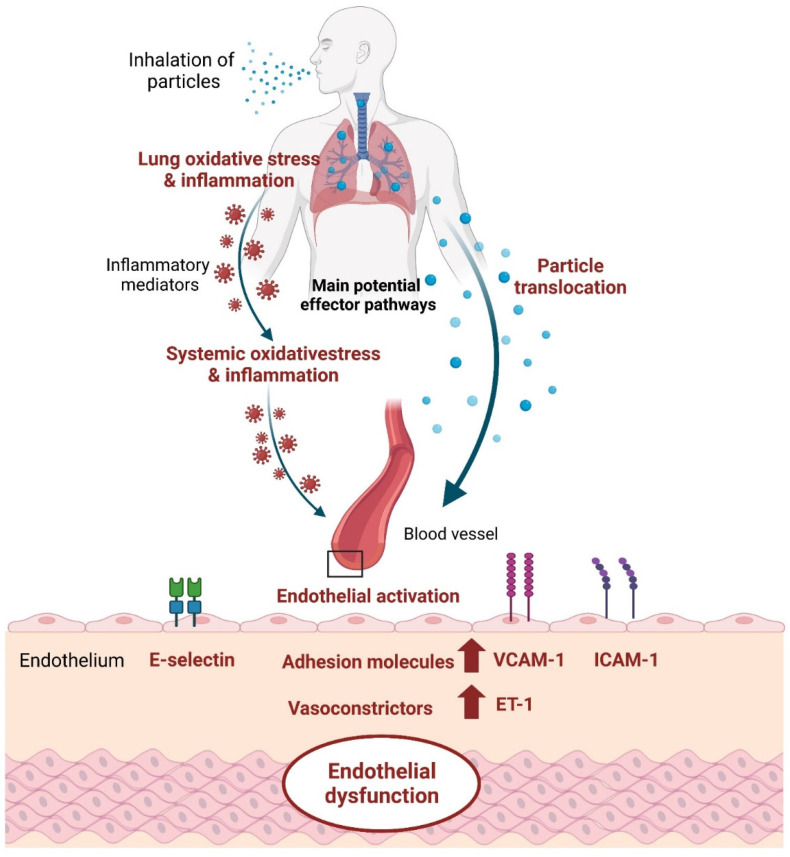
Biological mechanisms linking inhaled particles to endothelial dysfunction.

Indeed, endothelial dysfunction is strongly associated with almost all classical risk factors and is a key initiating event in the pathophysiological mechanisms of air pollution-mediated cardiovascular disease [19,53]. Previous studies have consistently shown that endothelial dysfunction is associated with atherosclerosis, atherosclerotic plaque rupture and thrombosis [54]. Several main pathophysiological insights into the mechanisms of PM_2.5_-mediated endothelial dysfunction and cardiovascular diseases have been proposed (Figure 3) [55]. The classical pathway was that inhaled PM activates pulmonary inflammation and generates reactive oxygen species, which release inflammatory mediators that further enter the circulation, induce dysfunction in distal tissues as blood vessels, and trigger cardiovascular events [14,56] (Figure 3). Systemic inflammation and oxidative stress may lead to the activation of endothelial cells, increased vascular permeability, and impairment of the endothelial vasomotor and vascular reparation function. It directly induces vascular injury and endothelial dysfunction, which involves increased systemic vasoconstrictors, including ET-1, tumor necrosis factor α, C-reactive protein, prostaglandin E2, and interleukin 1β, elevated pro-inflammatory factors and adhesion molecules such as interleukin 6, monocyte chemoattractant protein 1, E-selectin, VCAM-1, ICAM-1, soluble vascular cellular adhesion molecule 1 and soluble intercellular adhesion molecule 1sICAM-1, and pro-thrombosis, involving the von Willebrand factor and tissue factor [57,58]. Another proposed but controversial mechanism is that particulate constituents may also reach the systemic circulation via direct transfer from the lung (Figure 3) [58,59,60]. Nanoparticles in the circulation may interact with the vascular endothelium and directly trigger pro-inflammatory changes and oxidative stress in the vasculature and cardiac muscle [60,61]. Both mechanisms may lead to endothelial damage or endothelial dysfunction, which plays a key role in the development of atherosclerosis and acute myocardial ischemia [62,63].

ET-1 is a contractile peptide that constricts blood vessels [64,65] and is one of the most potent and persistent vasoconstrictor peptides with the greatest vasoconstrictive effect, and it is also the most abundant ET isomer in the human cardiovascular system [66,67]. The vasoconstriction effect of ET-1 is mainly modulated by its two receptors (types A and B) [68]. The type A receptor is primarily responsible for mediating vasoconstriction, cell proliferation, and hypertrophy, contributing to the regulation of vascular tone. In contrast, the type B receptor has a dual role. It induces vasodilation through the release of nitric oxide and prostacyclin when activated in endothelial cells, promoting blood flow and counteracting the vasoconstrictive effects. Moreover, type B receptors on vascular smooth muscle cells contribute to vasoconstriction. ET-1 and its receptor levels are elevated in a number of disease states associated with endothelial dysfunction [69,70]. These receptors are found on the surface of various cell types, including vascular smooth muscle cells and endothelial cells. The balance between vasoconstriction of ET-1 and vasodilation of nitric oxide (NO) is important to maintain normal vascular tone. Under normal conditions, NO has the function of anti-inflammation, inhibiting thrombosis, cell proliferation and maintaining vascular balance [71]. However, when ET-1 is maladjusted, it leads to endothelial dysfunction and an unbalanced release of vascular activity, leading to cardiovascular disease. After inhalation of particulate matter, oxidative stress in the body leads to the upregulation of ET-1 and down-regulation of NO production, further leading to an imbalance of endothelial function [72,73]. E-Selectin, ICAM-1 and VCAM-1 are adhesion molecules expressed on the surface of vascular endothelial cells, and they mediate the developmental mechanisms of atherosclerosis, such as the binding and recruitment of vascular endothelial cells to circulating leukocytes, and migration to the subcutaneous region [74,75]. When exposed to particulate air pollution, the production of reactive oxygen species is activated, and the levels of ICAM-1 and VCAM-1 are significantly elevated by activating the extracellular signal-regulated kinases, including protein kinase B and nuclear factor-kappa B pathways, and finally lead to endothelial dysfunction and vascular inflammation [24]. Therefore, the increases in these molecules caused by particulate air pollution and interference with normal body mechanisms may affect lung and cardiovascular functions [22]. Meanwhile, the inhalation of high concentrations of ambient particulate matter stimulates the bone marrow to release neutrophils, banded cells and monocytes into the body’s circulatory system. Thus, it expands the pool of white blood cells. The resulting systemic low-level inflammatory response leads to the upregulation of endothelial cell activation and adhesion molecules, which together with white blood cells participate in the formation of atherosclerotic plaques, resulting in adverse health outcomes [76]. The progression of atherosclerosis and the rupture of plaque are the mechanisms underlying the relationship between particulate air pollution and cardiovascular diseases [7,77]. Endothelial cell activation leads to an increased expression of adhesion molecules and inflammatory genes, a fundamental process in the development of atherosclerosis [42,66,67,71,78,79,80].

### 4.3. Main Findings from Subgroup Analysis

The results of the subgroup analysis for ICAM-1 indicated larger effect estimates for studies categorized as moderate quality, predominantly comprised of cross-sectional studies [21,39]. Notably, these studies primarily involved patients with type 2 diabetes, a subgroup recognized for heightened sensitivity to the adverse cardiovascular effects of air pollution [81,82]. In support of this finding, previous studies have found that exposure to PM_2.5_ is a potential risk factor for type 2 diabetes [83]. Addressing these differences through subgroup analyses, sensitivity analyses, and quality assessments, as described in our provided methodology, can help identify and account for whether methodological differences over included studies are sources of heterogeneity, ultimately improving the robustness of the meta-analysis findings.

The funnel plots of this meta-analysis showed that unpublished studies with negative results need to be added, indicating a slight publication bias (Figures S1 and S2). However, it is noteworthy that no publication bias was identified for ICAM-1 and VCAM-1 through Egger’s and Begg’s tests. Publication bias is evident when the addition of a missing study is required to balance asymmetry within a non-statistically significant region (white area) [31]. If missing references are added to the area of statistical significance (grey area), the asymmetry is due to other reasons rather than publication bias [84]. As can be seen from Figures S1 and S2, most of the included studies found that short-term exposure to PM_2.5_ might be a risk factor for endothelial dysfunction (risk ratio > 1), but negative associations (risk ratio <1) were also reported [39,42]. The heterogeneity observed between the included studies regarding the association between short-term exposure to PM_2.5_ and endothelial dysfunction might be attributed to variations in study quality, the demographic characteristics of participants, the control for confounding factors, and the composition of particulate air pollution.

### 4.4. Perspectives for Future Research and Policy

Research in the field of particulate air pollution and cardiovascular health should focus on understanding temporal and dose–response relationships, investigating mechanistic pathways through in-depth studies, identifying specific harmful components within particulate matter, exploring the unique impact of ultrafine particles, examining population vulnerability and health disparities, conducting longitudinal studies to track long-term effects, evaluating interventions’ effectiveness, and investigating global and regional variations in the association between particulate air pollution and biomarkers of cardiovascular health. These research directions aim to provide comprehensive insights into the nuanced aspects of the relationship, offering valuable information for regulatory measures, targeted interventions, and public health policies to mitigate cardiovascular risks associated with air pollution. Furthermore, we cannot find specific studies that distinguish the effects of natural and anthropogenic particulate matters on endothelial function factors. We do hope that future studies can fill this gap and answer whether the impact of particulate matter varies by source.

### 4.5. Limitations of This Review and Meta-Analysis 

In this meta-analysis which specifically focused on particulate air pollution and endothelial function, we found that short-term exposure to PM_2.5_ was positively associated with ICAM-1, VCAM-1. Although no significant correlation with ET-1 and E-selectin was observed, this may be due to the limitations of this study. Several limitations should be acknowledged. First, the inclusion of only English-published references from the retrieval databases may introduce language bias. Second, due to a limited number of eligible studies, meta-analyses for long-term exposures to PM_2.5_ and PM_10_ or short-term exposure to PM_10_ were not feasible. Third, variations in the adjustment of confounding factors across studies may contribute to the result deviation.

## 5. Conclusions

In conclusion, this meta-analysis revealed significant positive associations of short-term exposure to PM_2.5_ with ICAM-1 and VCAM-1, but not with ET-1 and E-selectin. However, due to the limited number of available studies, further research is needed to investigate the potential effects of particulate air pollution on endothelial function, especially the potential effects of long-term exposure. Nonetheless, this meta-analysis provides scientific evidence that short-term exposure to PM_2.5_ is associated with significant changes in markers of endothelial dysfunction, a potential mechanism linking particulate air pollution and the increased occurrence of cardiovascular diseases.

## Figures and Tables

**Table 1:** Characteristics of studies included in the meta-analysis.

Author	Study Duration	Location	Sample Size	Mean/Median Age	Female	Study Population	Exposure Assessment	Study Design	Outcome	Study Quality
Chen et al. (2022) [45]	2016–2019	USA	28	37	18	Healthy participants	Fixed monitoring site	Panel	ICAM-1, VCAM-1	High
Dai et al. (2016) [32]	1999–2010	USA	1565	74.9	0	Elderly	Fixed monitoring site	Cohort	ICAM-1, VCAM-1	High
Feng et al. (2021) [33]	2014–2016	China	73	23.3	48	Nonsmoking healthy adults	Fixed monitoring site	Panel	ICAM-1	High
Feng et al. (2021) [34]	2017–2018	China	88	21.1	54	Healthy college students	Fixed monitoring site	Panel	VCAM-1, ET-1	High
Finch et al. (2019) [35]	2019	USA	16	22	Unknown	Young healthy nonsmokers	Fixed monitoring site	Cohort	ET-1	High
Hajat et al. (2015) [15]	2000–2012	USA	7071	62	3740	Healthy adults	Model estimation	Cohort	ICAM-1, E-selectin,	High
Hu et al. (2021) [36]	2017–2018	China	44	23.3	14	Obese subjects and normal-weight subjects	Estimated individual exposure	Panel	ICAM-1	High
Li et al. (2017) [37]	2014	China	31	46	Unknown	Healthy nonsmoking participants	Fixed monitoring site	Quasi-experimental	ICAM-1, VCAM-1	High
Lin et al. (2019) [46]	2017–2018	China	31	22.87	18	Healthy college students	Fixed monitoring site	Randomized, double-blinded, and placebo-controlled trial	ET-1, E-selectin	High
Liu et al. (2017) [38]	2014	China	28	64	22	Elderly patients with COPD	Fixed monitoring site	Panel	ICAM-1, VCAM-1,	High
Mozzoni et al. (2022) [47]	2014–2016	Italy	295	34	295	Pregnant women	Model estimation	Cross-sectional	ICAM-1, VCAM-1	Moderate
O’Neill et al. (2007) [21]	1998–2002	USA	92	56.6	37	Type 2 diabetes	Fixed monitoring site	Cross-sectional	ICAM-1, VCAM-1	Moderate
Riggs et al. (2020) [39]	2011–2013	USA	100	48.1	56	General population	Fixed monitoring site	Cross-sectional	ICAM-1, VCAM-1, E-selectin	Moderate
Tong et al. (2015) [40]	2009–2010	USA	13	57.8	11	Healthy middle-aged human volunteers	Fixed monitoring site	Randomized controlled exposure study	ICAM-1, VCAM-1, ET-1	High
Wang et al. (2015) [41]	2013	China	36	66	Unknown	Healthy, nonsmoking college students	Fixed monitoring site	Panel	VCAM-1, ET-1	High
Wu et al. (2016) [42]	2010–2011	China	40	20.1	0	Healthy adults	Fixed monitoring site	Panel	ICAM-1, VCAM-1, ET-1, E-selectin	High
Wyatt et al. (2020) [43]	2017	USA	20	25.3	7	Healthy young volunteers	Fixed monitoring site	Randomized double-blind crossover study	ICAM-1, VCAM-1	Moderate
Zhang et al. (2020) [44]	2016	China	40	24.5	30	Healthy young college students	Fixed monitoring site	Panel	ICAM-1, VCAM-1	High

Abbreviations: ICAM-1, intercellular adhesion molecule-1; VCAM-1, vascular cell adhesion molecule-1; ET-1, endothelin-1; COPD, chronic obstructive pulmonary disease.

**Table 2:** Subgroup analysis for the association between short-term exposure to PM_2.5_ and ICAM-1.

Subgroup	Grouping Criteria	No. of Studies	Pooled %-Changes (95% CI)	*p*-Value	I-Squared	*p* for Heterogeneity	*p*-Value for Subgroup Difference
Study Area	North America	7	1.37 (0.03, 2.72)	0.046	54.74%	0.039	0.728
	Asia	7	1.66 (0.84, 2.48)	<0.001	45.34%	0.089	
	Europe	1	−0.70 (−6.94, 5.54)	0.825	—	—	
Participant	General population	12	1.27 (0.75, 1.79)	<0.001	40.00%	0.074	0.200
	Patients	3	2.80 (0.52, 5.08)	0.016	32.78%	0.226	
Sample Size	<1000	13	1.60 (0.90, 2.31)	<0.001	45.04%	0.039	0.795
	>1000	2	1.30 (−0.89, 3.49)	0.245	60.89%	0.110	
Age	<60	12	1.32 (0.79, 1.85)	<0.001	31.08%	0.143	0.442
	>60	3	2.22 (−0.01, 4.44)	0.051	74.81%	0.019	
Female Proportion	>50	8	1.33 (0.14, 2.52)	0.028	57.69%	0.021	0.180
	>50	6	1.65 (0.77, 2.53)	<0.001	0.00%	0.504	
	Unknown	1	8.29 (0.98, 15.59)	0.026	—	—	
Study Design	Panel	7	1.35 (0.79, 1.91)	<0.001	45.60%	0.087	0.700
	Others	5	1.77 (0.42, 3.11)	0.010	54.98%	0.064	
	Cross-sectional	3	4.38 (−4.83, 13.58)	0.351	55.78%	0.104	
Exposure Assessment	Fixed monitoring site	11	1.95 (1.05, 2.84)	<0.001	52.12%	0.022	0.143
	Model estimation	2	0.25 (−1.31, 1.81)	0.753	0.00%	0.757	
	Estimated individual exposure	2	1.06 (0.01, 2.12)	0.048	0.00%	0.586	
Study Quality	High	11	1.30 (0.81, 1.80)	<0.001	42.97%	0.063	0.113
	Moderate	4	2.70 (1.05, 4.35)	0.001	35.18%	0.201	

**Table 3:** Subgroup analysis for the association between short-term exposure to PM_2.5_ and VCAM-1.

Subgroup	Grouping Criteria	No. of Studies	Pooled %-Changes (95% CI)	*p*-Value	I-Squared	*p* for Heterogeneity	*p*-Value for Subgroup Difference
Area	North America	6	1.74 (0.65, 2.83)	0.002	63.86%	0.017	0.627
	Asia	6	2.26 (0.55, 3.96)	0.009	93.75%	<0.001	
	Europe	1	0.00 (−4.40, 4.40)	1.000	—	—	
Participant	General population	11	1.45 (0.50, 2.41)	0.003	87.27%	<0.001	0.183
	Patients	2	8.20 (−1.70, 18.09)	0.104	74.36%	0.048	
Sample Size	<1000	12	1.91 (0.73, 3.09)	0.001	88.33%	<0.001	0.591
	>1000	1	2.76 (−0.20, 5.73)	0.068	—	—	
Age	<60	10	1.73 (0.38, 3.07)	0.012	89.31%	<0.001	0.552
	>60	3	2.48 (0.39, 4.57)	0.020	80.43%	0.006	
Sex	>50	7	2.34 (1.06, 3.61)	<0.001	54.84%	0.039	0.824
	>50	4	1.96 (−0.57, 4.48)	0.128	83.41%	<0.001	
	Unknown	2	4.81 (−3.98, 13.60)	0.283	83.12%	0.015	
Design	Panel	6	1.96 (0.44, 3.47)	0.011	93.35%	<0.001	0.965
	Others	4	1.80 (0.71, 2.90)	0.001	53.75%	0.090	
	Cross-sectional	3	−0.01 (−16.87, 16.86)	0.999	83.61%	0.002	
Exposure Assessment	Fixed monitoring site	12	2.07 (0.91, 3.22)	<0.001	88.45%	<0.001	0.373
	Model estimation	1	0.00 (−4.40, 4.40)	1.000	—	—	
Study Quality	High	9	2.08 (0.79, 3.36)	0.002	90.30%	<0.001	0.826
	Moderate	4	0.90 (−9.52, 11.33)	0.865	75.56%	0.006	

## Data Availability

Data will be made available on request.

## Supplementary Materials

The following supporting information can be downloaded at: https://www.mdpi.com/article/10.3390/toxics12010076/s1, Table S1: Detailed search strategy of the meta-analysis; Table S2: Explanatory file for Effective Public Health Practice Project (EPHPP) Quality Assessment tool; Table S3: Quality assessment using the Effective Public Health Practice Project (EPHPP) Quality Assessment tool for the included studies; Table S4: Results of sensitivity analyses omitting one study each at a time for the associations between short-term exposure to PM_2.5_ and biomarkers of endothelial function; Table S5: Publication bias of the included studies for the association between short-term exposure to PM_2.5_ and biomarkers of endothelial function; Figure S1: Funnel plot of publication bias for the association between short-term exposure to PM_2.5_ and ICAM-1; Figure S2: Funnel plot of publication bias for the association between short-term exposure to PM_2.5_ and VCAM-1.
