# Supplementary material for: Association between Ambient Particulate Air Pollution and Soluble Biomarkers of Endothelial Function: A Meta-Analysis
Source: Toxics. 2024 Jan 15;12(1):76. doi: 10.3390/toxics12010076 (PMC10819696; doi:10.3390/toxics12010076)
Supplement: Supplementary file 1 [file toxics-12-00076-s001.zip › toxics-2779440-supplementary.pdf]

Supplementary Materials

Association between Ambient Particulate Air Pollution and Soluble Biomarkers of Endothelial Function: A Meta-Analysis

Kai Wang, Lei Lei, Ge Li, Yang Lan, Wanzhou Wang, Jiaqi Zhu, Qisijing Liu, Lihua Ren, Shaowei Wu

Table of contents

Table S1. Detailed search strategy of the meta-analysis.

Table S2. Explanatory file for Effective Public Health Practice Project (EPHPP) Quality Assessment tool.

Table S3. Quality assessment using the Effective Public Health Practice Project (EPHPP) Quality Assessment tool for the included studies.

Table S4. Results of sensitivity analyses omitting one study each at a time for the associations between short-term exposure to $PM_{2.5}$ and biomarkers of endothelial function.

Table S5. Publication bias of the included studies for the association between short-term exposure to $PM_{2.5}$ and biomarkers of endothelial function.

Figure S1. Funnel plot of publication bias for the association between short-term exposure to $PM_{2.5}$ and ICAM-1.

Figure S2. Funnel plot of publication bias for the association between short-term exposure to $PM_{2.5}$ and VCAM-1.

Table S1. Detailed search strategy of the meta-analysis.

Database	Keywords
**1. Pubmed**	**#1** "air pollution"[MeSH Terms] OR ("air"[All Fields] AND "pollution"[All Fields]) OR "air pollution"[All Fields] OR "particulate matter" OR "air pollutants" OR "particles" OR "PM2.5" OR "PM10"<br>**#2** ("ET-1"[All Fields] AND ("endotheline"[All Fields] OR "endothelins"[MeSH Terms] OR "endothelins"[All Fields] OR "endothelin"[All Fields])) OR "endothelin 1"[All Fields] OR "endothelin 1"[All Fields] OR "endothelin 1"[All Fields] OR "EDN1"[All Fields] OR "endothelial function"[All Fields] OR "endothelial dysfunction"[All Fields]<br>**#3** "E-selectin" OR "e selectin"[MeSH Terms] OR "e selectin"[All Fields] OR "e selectin"[All Fields] OR "ELAM-1" OR "E-sel" OR "CD62E" OR "sE-selectin" OR "soluble E-selectin" OR "endothelial adhesion receptors"<br>**#4** "intercellular adhesion molecule 1"[MeSH Terms] OR ("intercellular"[All Fields] AND "adhesion"[All Fields] AND "molecule 1"[All Fields]) OR "intercellular adhesion molecule 1"[All Fields] OR "icam 1"[All Fields] OR "Icam-1" OR "Intercellular cell adhesion molecule-1" OR "CD54" OR "icam-1" OR "intercellular adhesion molecule-1" OR "intercellular cell adhesion molecule 1" OR "sICAM-1" OR "endothelial-leukocyte adhesion molecule-1" OR "soluble ICAM-1" OR "cellular adhesion molecules"<br>**#5** "vascular cell adhesion molecule 1"[MeSH Terms] OR ("vascular"[All Fields] AND "cell"[All Fields] AND "adhesion"[All Fields] AND "molecule 1"[All Fields]) OR "vascular cell adhesion molecule 1"[All Fields] OR "vascular cell adhesion molecule 1"[All Fields] OR "VCAM-1" OR "Vascular Cell Adhesion Molecule-1" OR "vascular cell adhesion molecule-1" OR "CD106" OR "Vascular cell adhesion molecule 1" OR "vascular cell adhesion molecule 1" OR "soluble vascular cell adhesion molecule-1" OR "sVCAM-1" OR "soluble VCAM-1" OR "L-VCAM-1" OR "cellular adhesion molecules"<br>**#1 AND （#2 OR #3 OR #4 OR #5）**
**2. Embase**	('air pollution'/exp OR 'air pollution' OR 'particulate matter'/exp OR 'particulate matter' OR 'air pollutants'/exp OR 'air pollutants' OR 'particles' OR 'pm2.5'/exp OR 'pm2.5' OR 'pm10'/exp OR 'pm10') AND ('et-1' OR 'endotheline' OR 'endothelins'/exp OR 'endothelins' OR 'endothelin'/exp OR 'endothelin' OR 'endothelin 1'/exp OR 'endothelin 1' OR 'edn1' OR 'endothelial function' OR 'endothelial dysfunction'/exp OR 'endothelial dysfunction' OR 'e-selectin'/exp OR 'e-selectin' OR 'e selectin'/exp OR 'e selectin' OR 'elam-1'/exp OR 'elam-1' OR 'e-sel' OR 'cd62e' OR 'se-selectin' OR 'soluble e-selectin' OR 'endothelial adhesion receptors' OR 'intercellular adhesion molecule 1'/exp OR 'intercellular adhesion molecule 1' OR 'icam 1'/exp OR 'icam 1' OR 'intercellular cell adhesion molecule-1' OR 'cd54' OR 'icam-1'/exp OR 'icam-1' OR 'intercellular adhesion molecule-1'/exp OR 'intercellular adhesion molecule-1' OR 'intercellular cell adhesion molecule 1' OR 'sicam-1' OR 'endothelial-leukocyte adhesion molecule-1'/exp OR 'endothelial-leukocyte adhesion molecule-1' OR 'soluble icam-1' OR 'vcam-1'/exp OR 'vcam-1' OR 'vascular cell adhesion molecule-1'/exp OR 'vascular cell adhesion molecule-1' OR 'cd106' OR 'vascular cell adhesion molecule 1'/exp OR 'vascular cell adhesion molecule 1' OR 'soluble vascular cell adhesion molecule-1' OR 'svcam-1' OR 'soluble vcam-1' OR 'l-vcam-1' OR 'cellular adhesion molecules')
**3. Scopus**	1 TITLE-ABS-KEY ("air pollution" OR "particulate matter" OR "air pollutants" OR "particles" OR "pm2.5" OR "pm10")<br>2 TITLE-ABS-KEY ("et-1" OR "endotheline" OR "endothelins" OR "endothelin" OR "endothelin 1" OR "edn1" OR "endothelial function" OR "endothelial dysfunction")<br>3 TITLE-ABS-KEY ("e-selectin" OR "e selectin" OR "elam-1" OR "e-sel" OR "cd62e" OR "se-selectin" OR "soluble e-selectin" OR "endothelial adhesion receptors")<br>4 TITLE-ABS-KEY ("intercellular adhesion molecule 1" OR "icam 1" OR "icam-1" OR "intercellular cell adhesion molecule-1" OR "cd54" OR "icam-1" OR "intercellular adhesion molecule-1" OR "sicam-1" OR "endothelial-leukocyte adhesion molecule-1" OR "soluble icam-1" OR "cellular adhesion molecules")<br>5 TITLE-ABS-KEY ("vascular cell adhesion molecule 1" OR "vcam-1" OR "vascular

	cell adhesion molecule-1" OR "vascular cell adhesion molecule-1" OR "cd106" OR "vascular cell adhesion molecule 1" OR "vascular cell adhesion molecule 1" OR "soluble vascular cell adhesion molecule-1" OR "svcam-1" OR "soluble vcam-1" OR "l-vcam-1" OR "cellular adhesion molecules")
	1 AND (2 OR 3 OR 4 OR 5)
**4. Web of science**	TS=("air pollution" OR "particulate matter" OR "air pollutants" OR "particles" OR "PM2.5" OR "PM10") AND (TS=("ET-1" OR "endotheline" OR "endothelins" OR "endothelin" OR "endothelin 1" OR "EDN1" OR "endothelial function" OR "endothelial dysfunction") OR TS=("E-selectin" OR "e selectin" OR "ELAM-1" OR "E-sel" OR"CD62E" OR "sE-selectin" OR "soluble E-selectin" OR "endothelial adhesion receptors" OR TS=("intercellular adhesion molecule 1" OR "icam 1" OR "Icam-1" OR "Intercellular cell adhesion molecule-1" OR "CD54" OR "icam-1" OR "intercellular adhesion molecule-1" OR "sICAM-1" OR "endothelial-leukocyte adhesion molecule-1" OR "soluble ICAM-1" OR "cellular adhesion molecules") OR TS=("vascular cell adhesion molecule 1" OR "VCAM-1" OR "Vascular Cell Adhesion Molecule-1 " OR "vascular cell adhesion molecule-1" OR "CD106" OR "Vascular cell adhesion molecule 1" OR "vascular cell ad-hesion molecule 1" OR "soluble vascular cell adhesion molecule-1" OR "sVCAM-1" OR "soluble VCAM-1" OR "L-VCAM-1" OR " cellular adhesion molecules"))

Table S2. Explanatory file for Effective Public Health Practice Project (EPHPP) Quality Assessment tool.

Component ratings	Details
**A. Selection bias**	Good: (Q1 is 1) and (Q2 is 1).<br>Moderate: (Q1 is 1 or 2) and (Q2 is 2).<br>Poor: (Q1 is 3); or (Q2 is 3).
**Q1. Are the individuals selected to participate in the study likely to be representative of the target population?**	
1 Very likely	Randomly selected from a comprehensive list of individuals in the target population.
2 Somewhat likely	Referred from a source (e.g., clinic) in a systematic manner.
3 Not likely	Self-referred.
**Q2. What percentage of selected individuals agreed to participate?**	
1 80 - 100% agreement<br>2 60 – 79% agreement<br>3 Less than 60% agreement<br>4 Not applicable	Refers to the percentage of subjects in the control and intervention groups that agreed to participate in the study before they were assigned to intervention or control groups.
**B. Study design**	Good: (Q1 is 1).<br>Moderate: (Q1 is 2).<br>Weak: (Q1 is 3).
**Q1. Indicate the study design**	
1 Strong design	Randomized controlled trials (RCTs) and controlled clinical trials (CCTs).
2 Moderate design	Cohort or case-crossover designs.
3 Weak design	Cross-sectional or time-series designs.
**C. Confounders**	Good: (Q1 is 2) or (Q2 is 1).<br>Moderate: (Q1 is 1) and (Q2 is 2).<br>Poor: (Q1 is 1) and (Q2 is 3).
**Q1. Were there important differences between groups prior to the intervention?**	
1 Yes	Important differences were found.
2 No	No important differences were found.
**Q2. If yes, indicate the percentage of relevant confounders that were controlled (either in the design (e.g., stratification, matching) or analysis).**	
1 80-100%	Cross-sectional and cohort: meteorological variables and demographic characteristics.<br>Case-crossover and time-series: meteorological variables and time-effects.
2 60-79%	Cross-sectional and cohort: meteorological variables or demographic characteristics.<br>Case-crossover and time-series: meteorological variables or time-effects.
3 Less than 60% or none	Not controlled.
**D. Blinding**	Good: (Q1 is 2) and (Q2 is 2).<br>Moderate: (Q1 is 2) or (Q2 is 2).

	Poor: (Q1 is 1) and (Q2 is 1).
**Q1. Was (were) the outcome assessor(s) aware of the intervention or exposure status of participants?**	
1 Yes	The outcome assessors were aware of the exposure status of participants.
2 No	The outcome assessors were not aware of the exposure status of participants.
**Q2. Were the study participants aware of the research question?**	
1 Yes	The participants were aware of the exposure status of participants.
2 No	The participants were not aware of the exposure status of participants.
**E. Data collection methods**	Good: (Q1 is 1) and (Q2 is 1).<br>Moderate: (Q1 is 1) and (Q2 is 2).<br>Poor: (Q1 is 2).
**Q1. Were data collection tools shown to be valid?**	
1 Yes	The blood sample was detected by professionals and trained investigators.
2 No	The method for blood sample assay was not introduced.
**Q2. Were data collection tools shown to be reliable?**	
1 Yes	Methods had been acknowledged or with evidence from previous researches.
2 No	Methods were not clearly addressed.
**F. Withdrawals and drop-outs**	Good: (Q1 is 1) and (Q2 is 1).<br>Moderate: (Q2 is 2).<br>Poor: (Q2 is 3) or (Q1 is 2).<br>Not Applicable: (Q1 is 3) or (Q2 is 4).
**Q1. Were withdrawals and drop-outs reported in terms of numbers and/or reasons per group?**	
1 Yes	Describe both the numbers and reasons for withdrawals and drop-outs.
2 No	Either the numbers or reasons for withdrawals and drop-outs are not reported.
3 Not Applicable	No follow-up data.
**Q2. Indicate the percentage of participants completing the study. (If the percentage differs by groups, record the lowest).**	
1 80 -100%<br>2 60 - 79%<br>3 Less than 60%<br>4 Not Applicable	The percentage of participants completing the study refers to the % of subjects remaining in the study at the fi—l data collection period in all groups (i.e., control and intervention groups).

Overall ratings: 1) High—no “Poor” ratings. **2) Moderate—**one “Poor” rating. **3) Poor—**two or more “Poor” ratings.

Table S3. Quality assessment using the Effective Public Health Practice Project (EPHPP) Quality Assessment tool for the included studies.

**Author**	**Selection**	**Design**	**Confounder**	**Blinding**	**Data**	**Withdraw**	**Overall**
Chen et al. (2022)	Moderate	Moderate	High	Poor	High	High	High
Dai et al. (2016)	Moderate	Moderate	High	High	High	High	High
Feng et al. (2021)	Moderate	Moderate	High	High	High	High	High
Feng et al. (2021)	Moderate	Moderate	High	High	High	High	High
Finch et al. (2019)	Moderate	Moderate	High	High	High	Moderate	High
Hajat et al. (2015)	Moderate	Moderate	High	High	High	Moderate	High
Hu et al. (2021)	Moderate	Moderate	Moderate	High	High	High	High
Li et al. (2017)	Moderate	Moderate	High	High	High	High	High
Lin et al. (2019)	Moderate	High	High	High	High	High	High
Liu et al. (2017)	Moderate	Moderate	High	High	High	High	High
Mozzoni et al. (2022)	Moderate	Poor	High	High	High	—	Moderate
O'Neill et al. (2007)	Moderate	Poor	High	High	High	—	Moderate
Riggs et al. (2020)	Moderate	Poor	High	High	High	—	Moderate
Tong et al. (2015)	Moderate	High	High	High	High	High	High
Wang et al. (2015)	Moderate	Moderate	High	High	High	High	High
Wu et al. (2016)	Moderate	Moderate	High	High	High	High	High
Wyatt et al. (2020)	Moderate	Moderate	High	High	High	Poor	Moderate
Zhang et al. (2020)	Moderate	Moderate	High	High	High	High	High

Table S4. Results of sensitivity analyses omitting one study each at a time for the associations between short-term exposure to $PM_{2.5}$ and biomarkers of endothelial function.

Outcome	Pollutant	Study	Pooled %-changes (95%CI)	*P*-value	I-squared
ICAM-1	$PM_{2.5}$	Omitting Chen et al. (2022)	1.62% (0.97%, 2.28%)	<0.001	38.6%
ICAM-1	$PM_{2.5}$	Omitting Dai et al. (2016)	1.48% (0.79%, 2.16%)	<0.001	45.0%
ICAM-1	$PM_{2.5}$	Omitting Feng et al. (2021)	1.66% (0.85%, 2.47%)	<0.001	46.2%
ICAM-1	$PM_{2.5}$	Omitting Hajat et al. (2015)	1.68% (1.00%, 2.37%)	<0.001	42.7%
ICAM-1	$PM_{2.5}$	Omitting Hu et al. (2021)——Obese subjects	1.57% (0.83%, 2.32%)	<0.001	47.3%
ICAM-1	$PM_{2.5}$	Omitting Hu et al. (2021)——Normal weight subjects	1.67% (0.92%, 2.44%)	<0.001	45.5%
ICAM-1	$PM_{2.5}$	Omitting Li et al. (2017)	1.49% (0.85%, 2.13%)	<0.001	38.9%
ICAM-1	$PM_{2.5}$	Omitting Liu et al. (2017)	1.30% (0.80%, 1.79%)	<0.001	30.2%
ICAM-1	$PM_{2.5}$	Omitting Mozzoni et al. (2022)	1.57% (0.91%, 2.24%)	<0.001	46.4%
ICAM-1	$PM_{2.5}$	Omitting O'Neill et al. (2007)	1.54% (0.88%, 2.20%)	<0.001	46.9%
ICAM-1	$PM_{2.5}$	Omitting Riggs et al. (2020)	1.52% (0.87%, 2.18%)	<0.001	37.5%
ICAM-1	$PM_{2.5}$	Omitting Tong et al. (2015)	1.63% (0.91%, 2.36%)	<0.001	46.3%
ICAM-1	$PM_{2.5}$	Omitting Wu et al. (2016)	1.61% (0.89%, 2.33%)	<0.001	46.9%
ICAM-1	$PM_{2.5}$	Omitting Wyatt et al. (2020)	1.32% (0.82%, 1.82%)	<0.001	41.2%
ICAM-1	$PM_{2.5}$	Omitting Zhang et al. (2020)	1.50% (0.80%, 2.22%)	<0.001	46.0%
ICAM-1	$PM_{2.5}$	Pooled estimate	1.55% (0.89%, 2.22%)	<0.001	43.3%
—	—	—	—	—	—
VCAM-1	$PM_{2.5}$	Omitting Chen et al. (2022)	1.91% (0.80%, 3.03%)	0.001	88.4%
VCAM-1	$PM_{2.5}$	Omitting Dai et al. (2016)	1.93% (0.73%, 3.14%)	0.001	88.3%
VCAM-1	$PM_{2.5}$	Omitting Feng et al. (2021)	1.93% (0.64%, 3.24%)	0.003	79.6%
VCAM-1	$PM_{2.5}$	Omitting Li et al. (2017)	1.78% (0.73%, 2.83%)	0.001	87.7%
VCAM-1	$PM_{2.5}$	Omitting Liu et al. (2017)	1.60% (0.60%, 2.62%)	0.002	87.1%
VCAM-1	$PM_{2.5}$	Omitting Mozzoni et al. (2022)	2.09% (0.91%, 3.27%)	<0.001	88.4%
VCAM-1	$PM_{2.5}$	Omitting O'Neill et al. (2007)	1.80% (0.75%, 2.87%)	0.001	87.6%
VCAM-1	$PM_{2.5}$	Omitting Riggs et al. (2020)	2.06% (0.95%, 3.18%)	<0.001	87.8%
VCAM-1	$PM_{2.5}$	Omitting Tong et al. (2015)	2.15% (0.92%, 3.39%)	0.001	88.5%
VCAM-1	$PM_{2.5}$	Omitting Wang et al. (2015)	2.18% (0.89%, 3.50%)	0.001	88.5%
VCAM-1	$PM_{2.5}$	Omitting Wu et al. (2016)	2.25% (1.29%, 3.21%)	<0.001	70.9%
VCAM-1	$PM_{2.5}$	Omitting Wyatt et al. (2020)	2.07% (0.77%, 3.39%)	0.002	88.4%
VCAM-1	$PM_{2.5}$	Omitting Zhang et al. (2020)	1.93% (0.74%, 3.13%)	0.001	88.3%
VCAM-1	$PM_{2.5}$	Pooled estimate	1.97% (0.86%, 3.08%)	<0.001	87.4%
—	—	—	—	—	—
ET-1	$PM_{2.5}$	Omitting Feng et al. (2021)	-0.12% (-7.20%, 7.49%)	0.974	83.4%
ET-1	$PM_{2.5}$	Omitting Finch et al. (2019)	1.42% (0.53%, 2.31%)	0.002	70.3%
ET-1	$PM_{2.5}$	Omitting Lin et al. (2019)	-0.83% (-5.67%, 4.27%)	0.746	84.5%
ET-1	$PM_{2.5}$	Omitting Tong et al. (2015)	-0.09% (-7.17%, 7.54%)	0.981	85.5%
ET-1	$PM_{2.5}$	Omitting Wang et al. (2015)	-0.19% (-7.21%, 7.37%)	0.960	84.9%

ET-1	$PM_{2.5}$	Omitting Wu et al. (2016)	0.20% (-6.99%, 7.96%)	0.957	80.0%
ET-1	$PM_{2.5}$	Pooled estimate	0.22% (-4.94%, 5.65%)	0.936	82.70%
—	—	—	—	—	—
E-selectin	$PM_{2.5}$	Omitting Hajat et al. (2015)	3.98% (-2.61%, 11.01%)	0.243	84.0%
E-selectin	$PM_{2.5}$	Omitting Lin et al. (2019)	1.30% (-1.87%, 4.58%)	0.425	54.8%
E-selectin	$PM_{2.5}$	Omitting Riggs et al. (2020)	2.71% (-1.57%, 7.16%)	0.218	82.9%
E-selectin	$PM_{2.5}$	Omitting Wu et al. (2016)	4.87% (0.74%, 9.17%)	0.020	66.8%
E-selectin	$PM_{2.5}$	Pooled estimate	3.21% (-0.90%, 7.49%)	0.128	76.50%
—	—	—	—	—	—

Table S5. Publication bias of the included studies for the association between short-term exposure to $PM_{2.5}$ and biomarkers of endothelial function.

**Outcome**	**No. of estimates**	**Begg's *P*-value**	**Egger's *P*-value**
ICAM-1	15	0.125	0.213
VCAM-1	13	1.00	0.290
ET-1	6	—	—
E-selectin	4	—	—

Note: Begg's test and Egger's tests were unable to be conducted for some associations due to the small numbers of studies included.

Figure S1. Funnel plot of publication bias for the association between short-term exposure to $PM_{2.5}$ and ICAM-1.

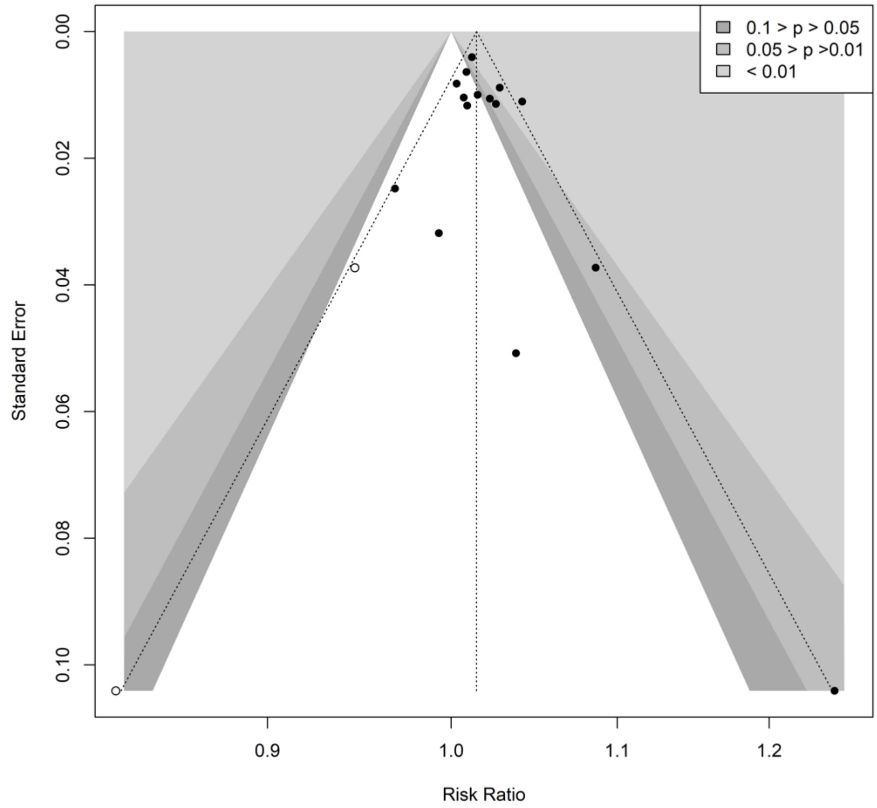

Note: Solid dots indicate the studies included in this meta-analysis, and hollow dots indicate missing literature added using the trim-and-fill method.

Figure S2. Funnel plot of publication bias for the association between short-term exposure to $PM_{2.5}$ and VCAM-1.

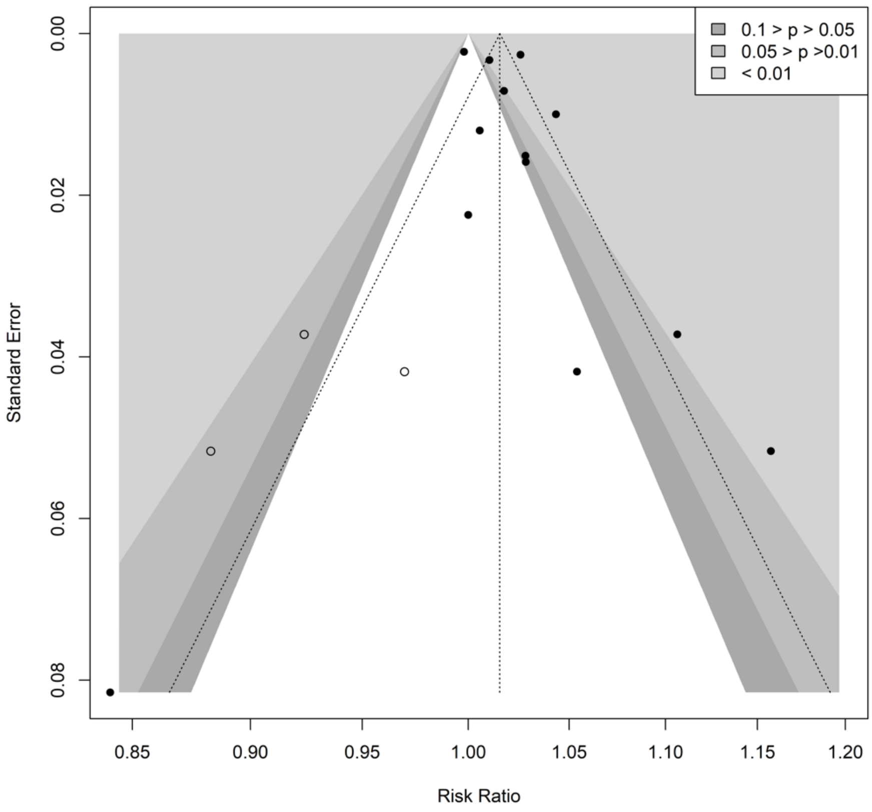

Note: Solid dots indicate the studies included in this meta-analysis, and hollow dots indicate missing literature added using the trim-and-fill method.
